# *ConR*: An R package to assist large‐scale multispecies preliminary conservation assessments using distribution data

**DOI:** 10.1002/ece3.3704

**Published:** 2017-12-16

**Authors:** Gilles Dauby, Tariq Stévart, Vincent Droissart, Ariane Cosiaux, Vincent Deblauwe, Murielle Simo‐Droissart, Marc S. M. Sosef, Porter P. Lowry, George E. Schatz, Roy E. Gereau, Thomas L. P. Couvreur

**Affiliations:** ^1^ Institut de Recherche pour le Développement (IRD) Université Montpellier, UMR DIADE Montpellier France; ^2^ Evolutionary Biology and Ecology Unit Faculté des Sciences Université Libre de Bruxelles Bruxelles Belgium; ^3^ French Foundation for Research on Biodiversity (FRB) through its Center for Synthesis and Analysis of Biodiversity data (CESAB) program Domaine du Petit Arbois Aix‐en‐Provence France; ^4^ Africa and Madagascar Department Missouri Botanical Garden St. Louis MO USA; ^5^ Botanic Garden Meise Meise Belgium; ^6^ Herbarium et Bibliothèque de Botanique Africaine Faculté des Sciences, Université Libre de Bruxelles Bruxelles Belgium; ^7^ Institut de Recherche pour le Développement (IRD) UMR AMAP Montpellier France; ^8^ Plant Systematics and Ecology Laboratory Higher Teachers Training College University of Yaoundé I Yaoundé Cameroon; ^9^ Center for Tropical Research Institute of the Environment and Sustainability University of California Los Angeles CA USA; ^10^ International Institute of Tropical Agriculture Yaoundé Cameroon; ^11^ Institut de Systématique Évolution et Biodiversité (ISYEB) Unité Mixte de Recherche 7205 (Centre National de la Recherche Scientifique/Muséum National d'Histoire Naturelle/École Pratique des Hautes Études/Université Pierre et Marie Curie) Muséum national d'Histoire naturelle Sorbonne Universités Paris France

**Keywords:** area of occupancy, criterion B, distribution range, extent of occurrence, IUCN, location, preliminary status, threatened taxa

## Abstract

The Red List Categories and the accompanying five criteria developed by the International Union for Conservation of Nature (IUCN) provide an authoritative and comprehensive methodology to assess the conservation status of organisms. Red List criterion B, which principally uses distribution data, is the most widely used to assess conservation status, particularly of plant species. No software package has previously been available to perform large‐scale multispecies calculations of the three main criterion B parameters [extent of occurrence (EOO), area of occupancy (AOO) and an estimate of the number of locations] and provide preliminary conservation assessments using an automated batch process. We developed *ConR*, a dedicated R package, as a rapid and efficient tool to conduct large numbers of preliminary assessments, thereby facilitating complete Red List assessment. *ConR* (1) calculates key geographic range parameters (AOO and EOO) and estimates the number of locations *sensu* IUCN needed for an assessment under criterion B; (2) uses this information in a batch process to generate preliminary assessments of multiple species; (3) summarize the parameters and preliminary assessments in a spreadsheet; and (4) provides a visualization of the results by generating maps suitable for the submission of full assessments to the IUCN Red List. *ConR* can be used for any living organism for which reliable georeferenced distribution data are available. As distributional data for taxa become increasingly available via large open access datasets, *ConR* provides a novel, timely tool to guide and accelerate the work of the conservation and taxonomic communities by enabling practitioners to conduct preliminary assessments simultaneously for hundreds or even thousands of species in an efficient and time‐saving way.

## 1 INTRODUCTION

As we attempt to address the modern biodiversity crisis, assessing the conservation status of species has become an invaluable tool for biodiversity conservation. Evaluating threat based on the Red List Categories and Criteria of the International Union for Conservation of Nature (IUCN, 2012) is an authoritative, comprehensive and widely used approach in conservation biology (Rodrigues, Pilgrim, Lamoreux, Hoffmann, & Brooks, 2006). Indeed, many decisions made by governments, natural resource managers, and conservation planners (Rodrigues et al., 2006) rely (often solely) on the “Red List” published by IUCN (http://www.iucnredlist.org/). For example, programs such as Important Bird Areas (IBA), Important Plant Areas (IPA, Anderson, 2002) or Tropical Important Plant Areas (TIPA, Darbyshire et al., 2017) all rely directly on threat assessments based on IUCN criteria. In parallel, there is also an urgency in listing threatened species in the near future. This is, for example, the case of Target 2 of the Global Strategy for Plant Conservation (GSPC) of the United Nation's Convention on Biological Diversity, which calls for assessing the conservation status of all known plant species by 2020 (https://www.cbd.int/gspc/targets.shtml).

However, as of 2016, the Red List included assessments of just 21,898 plant species (IUCN Standards and Petitions Subcommittee, 2016), ca. 6.2% of the estimated global total (~352,000 flowering plant species; Paton et al., 2008). The ThreatSearch database (www.bgci.org/threat_search.php) documents the conservation assessments of ca. 150,000 taxa, including assessments at the species and infra‐specific levels based on both older or current IUCN criteria; preliminary, global or regional assessments; and assessments based on other non‐IUCN criteria. Thus, ThreatSearch represents an uncritical, high‐end estimate of the total number of plant taxa assessed to date. Hence, over the last three decades, progress toward this target has been slow largely because the process of performing and publishing full Red List assessments is time‐consuming. Accelerating global conservation assessments is urgently needed (Krupnick, Kress, & Wagner, 2009; Miller et al., 2012). While alternative methods have been developed to streamline and simplify large‐scale conservation assessments (e.g., Krupnick et al., 2009; Miller et al., 2012; Ocampo‐Peñuela, Jenkins, Vijay, Li, & Pimm, 2016; Ter Steege et al., 2015), none are based on the theoretical framework provided by IUCN, and they thus have little immediate impact for concrete conservation actions.

The International Union for Conservation of Nature employs five complementary criteria (A, B, C, D and E) under which a species can be evaluated, and, when not already extinct, assessments assign species to three threatened categories (Critically Endangered (CR); Endangered (E); VU (Vulnerable)), or otherwise to LC (Least Concerned), NT (Near Threatened) or DD (Data Deficient, when insufficient data are available). Among these five criteria, criterion B is the most widely used. For example, in 2007, almost half of all organisms whose status was published on the IUCN Red List were assessed solely based on criterion B (Gaston & Fuller, 2009). Unlike the others, Criterion B is suitable for estimating conservation status even when the distribution of a taxon is only known from georeferenced herbarium or museum collections and with limited information on local threats and potential continuing decline (Schatz, 2002), and it plays a prominent role in describing global trends in extinction risk. Even though some have suggested that Criterion B is the most misapplied of the five (IUCN Standards and Petitions Subcommittee 2016, p. 62), it nevertheless has the significant advantage of allowing assessments to be undertaken using distribution data only (Schatz, 2002), which are in many cases the only information available (in contrast, for example, to abundance data).

Assessing the conservation status of taxa under IUCN Red List criterion B (IUCN, 2012; IUCN Standards and Petitions Subcommittee 2016) nevertheless presents particular challenges based on recorded primary occurrences (typically obtained by compiling herbarium/museum records). Criterion B involves two subcriteria (B1 and B2), which reflect two different kinds of geographic range size estimates [subcriterion B1 is based on extent of occurrence (EOO) while B2 is based on area of occupancy (AOO)], and three additional conditions (a, b and c) that describe aspects of the biology and potential decline of the taxon as a result of the impact of threats. Threshold levels for at least one subcriterion and two conditions must be met for a taxon to be assigned a threatened conservation status (see Table 1).

**Table 1:** Subcriteria and conditions used in *ConR* to estimate preliminary conservation status under IUCN criterion B

Subcriteria/conditions		Method in *ConR*
B1	Extent of occurrence (EOO)	Convex hull or alpha hull
B2	Area of occupancy (AOO)	Grid of user‐selected resolution superimposed on range‐wide occurrences
(a)	Range severely fragmented	Not implemented
	OR	Grid of user‐selected resolution superimposed on range‐wide occurrences and level of threat estimated by mapping protected areas (see vignette package)
	Number of locations	
(b) (iii)	Continuing decline observed, inferred or projected of habitat quality	Assumed to be true for taxa with a limited distribution because of the current threat on habitat, which most likely implies a future decline of the quality of habitat
(b) (i, ii, iv, v)	Continuing decline observed, inferred or projected of EOO, AOO, number of locations/subpopulations, number of mature individuals	Not implemented
(c) (i to iv)	Extreme fluctuations of various descriptors of geographic range	Not implemented

### 1.1 Extent of occurrence

Extent of occurrence (EOO) is defined as “the area contained within the shortest continuous imaginary boundary that can be drawn to encompass all the known, inferred or projected sites of present occurrence of a taxon, excluding cases of vagrancy (IUCN, 2012).” EOO is generally measured by a minimum convex polygon, or convex hull, defined as “the smallest polygon in which no internal angle exceeds 180° and which contains all the sites of occurrence (IUCN, 2012).” Alternatively, in certain situations, EOO can be calculated as an alpha Hull (see IUCN Standards and Petitions Subcommittee 2016).

### 1.2 Area of occupancy

The Area of occupancy (AOO) is defined as “the area within its ‘extent of occurrence’ that is occupied by a taxon, excluding cases of vagrancy (IUCN, 2012).” AOO differs from EOO (see above) as it reflects the fact that a taxon will not usually occur throughout its EOO, that is, there will be areas where the taxon is absent, including (unsuitable areas). The AOO will be a function of the scale or grid cell size at which it is measured, and which should reflect relevant biological aspects of the taxon. For example, the impact of a threat is not identical if we consider tree or herb species.

### 1.3 Location

A “location” is defined as “a geographically or ecologically distinct area in which a single threat can rapidly affect all individuals of the taxon present (IUCN, 2012).” Thus, the size of a location depends on the threat (mining, deforestation, poaching, etc.). EOO and AOO, the two main parameters of Criterion B, can be generated automatically (Table 1). However, assessing the number of locations requires contextual information about threats. This information, which is usually obtained from field observations, expert knowledge, and/or precise data on the size and nature of a taxon's range (e.g., continuous vs. severely fragmented), can thus only be applied properly using a “taxon‐by‐taxon” process to obtain a fully informed IUCN Red List assessment.

### 1.4 Subpopulations

“Subpopulations” are defined as “geographically or otherwise distinct groups in the population between which there is little demographic or genetic exchange (IUCN, 2012; Rivers, Bachman, Meagher, Lughadha, & Brummitt, 2010).” Although the number of subpopulations is not directly taken into account for assessments based on criterion B, this information is requested during the submission process to the IUCN Red List.

Below, we describe *ConR*, an R package to generate batch preliminary assessments of conservation status following the IUCN guidelines using multiple species datasets based on Criterion B. *ConR* makes it possible to: (1) calculate or estimate the key parameters needed for an assessment under criterion B; (2) generate preliminary assessments of multiple species using a batch process; and (3) summarize the estimated parameters and preliminary assessments in a spreadsheet and spatially visualize the results on generated maps. *ConR* implements a novel method to approximate the number of “locations” *sensu* IUCN, one of the key Criterion B parameters (see below).

## 2 THE *ConR* PACKAGE

*ConR* allows users to estimate the above parameters automatically for any list of taxa and then assigns each taxon to a preliminary IUCN threat category according to Criterion B. These preliminary assessments are based on calculations of EOO and AOO and an estimate of the number of locations for each taxon [condition (a); Table 1]. The rationale behind *ConR* is to facilitate preliminary conservation assessments based on large sets of species distribution data. In order to achieve this, *ConR* uses a number of assumptions about certain parameters or future trends that would have to be inferred on a taxon‐by‐taxon basis for a full IUCN assessments. The results obtained from *ConR* therefore should not be taken as full or definitive Red List IUCN assessments.

Under Criterion, B, the assessment of a taxon is based on the calculation of its EOO (B1) and/or AOO (B2). In addition, at least two of the following conditions must be taken into consideration: (1) the number of locations; (2) continuing decline of different aspects of its distribution (EOO, AOO, number of locations, etc.); and (3) extreme fluctuation of certain aspects of the taxon's distribution (Table 1). Calculation of the two key range parameters, EOO and AOO, can be easily automated either using a taxon‐by‐taxon approach, as provided for by the web service *GeoCAT* (Bachman, Moat, Hill, de la Torre, & Scott, 2011), or in batch mode, for example in other R packages such as *speciesgeocodeR* (Töpel et al., 2017) or RED (https://CRAN.R-project.org/package=red; see Table 2).

**Table 2:** Features of various currently available programs that estimate parameters used for preliminary conservation status assessments following the IUCN guidelines. *GeoCAT* (Bachman et al., 2011); *speciesgeocodeR* (Töpel et al., 2017) and RED (https://CRAN.R-project.org/package=red)

Features	Definition (IUCN Standards and Petitions Subcommittee, 2016)	*GeoCAT*	*speciesgeocodeR*	RED	*ConR*
Batch or multispecies estimates		No	Yes	Yes	Yes
Extent of occurrence (EOO) calculation	Intended to “measure the degree to which risks from threatening factors are spread spatially across the taxon's geographical distribution”	Yes	Yes	Yes	Yes
Area of occupancy (AOO) calculation	Area within a taxon's EOO that is occupied by the taxon	Yes	Yes	Yes	Yes
Estimate of number of subpopulations	“Geographically or otherwise distinct groups in the population between which there is little demographic or genetic exchange”	No	No	No	Yes
Estimate of number of locations	“Geographically or ecologically distinct area in which a single threatening event can rapidly affect all individuals of the taxon present”	No	No	No	Yes
IUCN ready maps		No	No	Yes	Yes

However, none of these packages are designed to estimate the number of locations, a fact that hinders their utility in assigning taxa to a threat category under Criterion B. The notion of “location” remains a complex and sometimes confusing concept. It has been interpreted in many different ways depending on the type of organism studied, the general landscape in which a taxon occurs and the type of threat to its populations. In *ConR* we have, for the first time, attempted to estimate the number of locations automatically so that it can be calculated simultaneously for a large number of taxa. This automation comes with a number of assumptions detailed below.

The number of locations for each taxon can be approximated using two complementary approaches in *ConR*. First, a grid with cells of a chosen size is overlaid on taxa occurrences and the number of locations is estimated by the number of occupied cells. The grid cell size must be defined by the user and should represent the scale at which subpopulations are equally affected by a given threat. For example, a cell size of 10 km² may be considered a good estimate of the scale at which a particular serious threat event such as mining could equally affect individuals of a given taxon (Durán et al. 2013). The user can choose a fixed cell size across the whole multispecies dataset (e.g. 10 km²) or can use a species‐specific sliding scale approach (Rivers et al., 2010). In the latter approach, cell size is defined as 1/*x* of the maximum interoccurrence distance, where *x* is the maximum distance between two occurrences (e.g. 5% (0.05) of the max distance between the known occurrences). In both cases, the cell grid is overlaid on the total distribution of the taxon in a way that results in the minimum number of estimated locations. Finally, as cell size is user defined, alternative estimates of the scale at which a given threat operates can be compared.

In the second approach, *ConR* integrates information about protected areas (PAs). The underlying rationale for this is that subpopulations within a PA will not be treated in the same way as those located outside a PA. *ConR* deals with PA in two ways (*method_protected_area* argument). First, occurrences within a given PA are assumed to fall within the same location irrespective of the size of the PA (“no_more_than_one”). Subpopulations within a PA are thus assumed to be subject to the same threat. For example, protected area downgrading, downsizing, or degazetting (PADDD), a common occurrence throughout the tropics (Mascia et al., 2014), is assumed to affect all individuals within that PA in the same manner (one threat, PADDD affecting the whole of the PA). Similarly, if illegal exploitation takes place in a PA, it is assumed that this could potentially impact all individuals of the taxon. Second, the number of locations situated within PAs is estimated separately from those occurring outside PAs (“other”), thereby decoupling the estimation of locations within and outside of PAs. Thus, two individuals could be geographically close (separated by less than the selected grid cell size) but in separate locations, one in a PA and the other not, because the nature of the threat is not the same.

Both approaches used by *ConR* to estimate the number of locations (*Cell_size_locations* and *method_protected_area*) are customizable by the user, who can decide what cell size to use, whether or not to include PAs, and if so, how to take them into account. Also, if alternative shapefiles are available for the PAs, the user can select which one to use, such as the World Database on Protected Areas (WDPA, https://www.protectedplanet.net).

For each preliminary assessment, in addition to estimating the “number of locations” condition, at least one of the two remaining conditions relating to the future trend of a taxon's distribution or structure must be taken into consideration: continuing decline and/or extreme fluctuation. *ConR* assumes by default a continuing future decline in habitat quality [condition (b) (iii), Table 1]. While this assumption might appear be an oversimplification, it would seem to be valid in most cases. The validity of this assumption is also intuitively acknowledged by the IUCN guidelines, which recognize a criterion for assessing threat status specifically on the basis of very small or restricted populations (Criterion D). This assumption is also reasonable when one considers that wilderness areas are in rapid decline throughout the world, especially in the tropics (Watson et al., 2016), suggesting that future decline may be anticipated for any given range‐restricted species.

Finally, *ConR* also provides an estimate of the number of subpopulations of a taxon by implementing a circular buffer method (Resol_sub_pop in km²). This buffer is user defined and can be adapted to different groups of taxa depending on their different dispersal characteristics but also gene flow (if known).

## 3 *ConR* FEATURES

**Figure 1:**
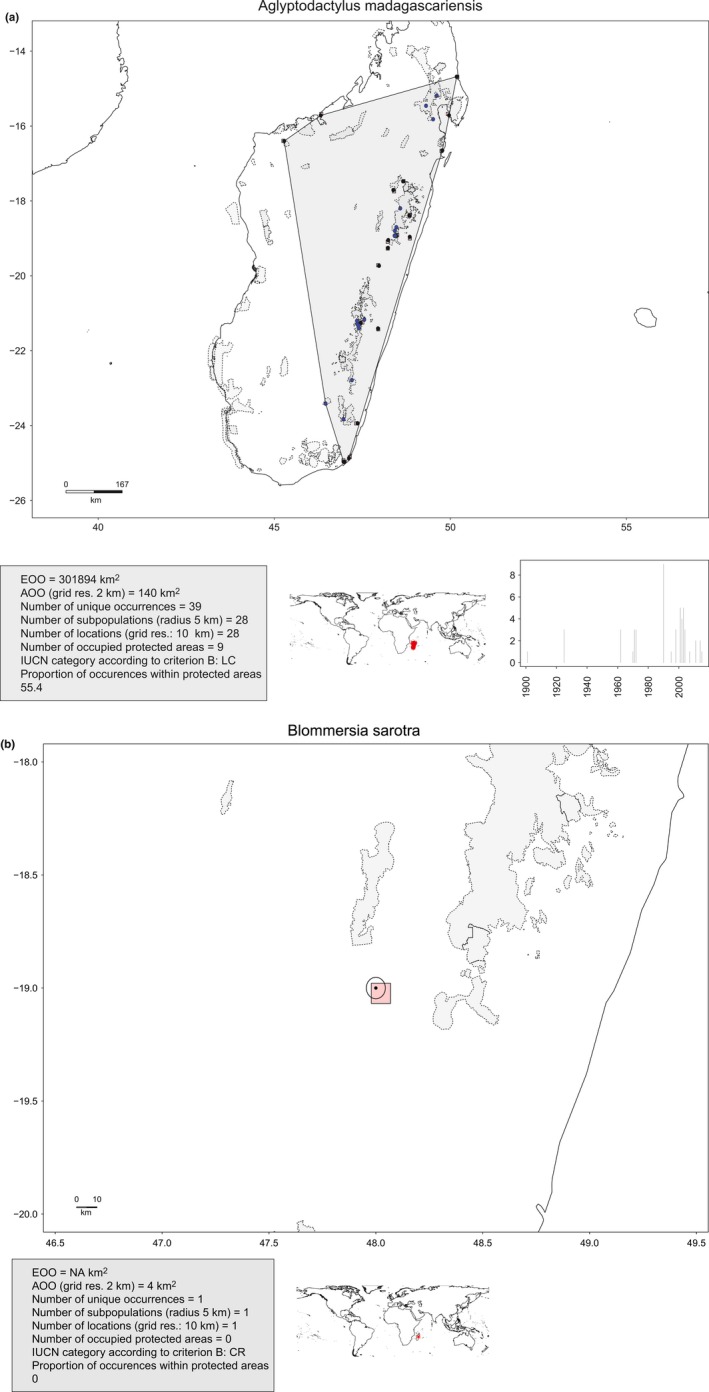
Two examples of map outputs generated by the *IUCN.eval* function of *ConR* for two species of Malagasy amphibians that were chosen from the example dataset: (a) *Aglyptodactylus madagascariensis* and (b) *Blommersia sarotra*. The top main inset shows a map of the region concerned with the occurrences of the species shown as black dots (records situated outside protected areas) and as blue dots (within protected areas). The delimitation of locations is shown by pink squares and of subpopulations by circles. For the species in (a), the convex hull used for calculating the EOO is shown for the first species as a gray polygon. For the species in (b), the EOO was not calculated because it has a single known occurrence. The bottom left gray inset summarizes all the information calculated by the *IUCN.eval* function. The bottom middle inset situates the species’ distribution on a world map, with red crosses representing its occurrences. Finally, the bottom right inset shows the number of collections per year as a bar plot, when these data are available [e.g., in (a)]. In both examples, the status of the preliminary assessments provided by *ConR* would be the same as those from the formal assessments

*ConR* includes four functions, two sample occurrence datasets, and two sample shapefile datasets. All functions operate on a mandatory single data frame providing taxon occurrences and on optional user‐provided shapefiles of land/sea and protected area limits. Occurrence data and shapefiles must be provided using the WGS84 reference coordinate system. The input data frame requires three mandatory fields: latitude and longitude (in decimal degrees), and taxon name. The collection year can also be added, thereby allowing graphic visualization of a taxon's collecting history (Figure 1). Additional information, such as higher taxonomic rank, can also be provided. By default, *ConR* saves all results in the user's R working directory. A step by step tutorial (R vignette) describing all options is provided as supplementary material and on the CRAN website.

### 3.1 *IUCN.eval*

This is the main *ConR* function, which provides values for all parameters, including EOO, AOO and an estimate of number of locations, needed for assessing the preliminary conservation status of taxa based on selected conditions and subcriteria of criterion B (Table 1). All options are flexible and can be user defined. The number of locations can be estimated using a fixed or sliding grid approach (Rivers et al., 2010). In addition, PA information can also be taken into account if an appropriate PA shapefile is provided (see above).

The output is a table in a comma‐separated values (CSV) file summarizing the different parameters calculated for each taxon. Besides all of the parameters calculated or estimated, *ConR* explicitly assigns a preliminary threat category for each taxon under the validated criteria (B1 and/or B2). In addition, one can see the threat assignments based either on EOO (B1) or AOO (B2). The output provides the user with a clear presentation of all the basic information needed to undertake a full IUCN assessment. Results can also be visualized graphically (see Figure 1) via the argument *DrawMap*. A folder is automatically generated with a figure for each taxon. Figures are generated in PNG format, which minimizes output size for large‐scale multitaxon studies (each map is on average 100 kb). The figure for each taxon contains a summary of the estimated parameters and the threat assignment, along with a distribution map. If PA information was included, the map also depicts the distribution of PAs as well as occurrences within (blue dots) or outside (black dots) them (see Figure 1). This map can be used for the submission of a formal assessment to the IUCN.

### 3.2 *EOO.computing*

The *EOO.computing* function calculates EOO. It operates with a minimum of three unique occurrences; otherwise, it returns “NA”. In *ConR*, EOO can be estimated either using a “convex.hull” or an “alpha.hull” method, as recommended by IUCN Standards and Petitions Subcommittee (2016). Cropping of unsuitable areas (e.g., water bodies) before the calculation of EOO is available via the argument *exclude.area*. It is important to note, however, that excluding areas from the EOO calculation and the estimate EOO with alpha Hull are explicitly discouraged by the IUCN guidelines (2016) when using Criterion B. Finally, if the EOO is less than the AOO, then the EOO is set to be equal to the AOO, as recommended by the IUCN Standards and Petitions Subcommittee (2016).

In the very infrequent case that occurrences form a straight segment, the EOO will be zero, representing an underestimate of its surface (IUCN Standards and Petitions Subcommittee 2016). In this specific case, *ConR* outputs a warning. The EOO is then estimated using a different method: A polygon is built by adding a buffer of a predefined size of 0.1° to the segment, which can be adjusted by the argument *buff.alpha*. Also, the EOO cannot be computed when there are less than three unique occurrences; a warning is returned in such case. Finally, it should be noted that the way in which *ConR* estimates the EOO may be biased for species with wide distributions and cannot be applied to species whose distribution spans the 180th meridian (see R documentation).

### 3.3 *subpop.comp*

This function estimates the number of subpopulations using the circular buffer method (Rivers et al., 2010). Each unique occurrence is buffered with a circle of a defined radius and overlapping circles are merged to form a single subpopulation, while nonoverlapping circles are considered to represent separate subpopulations. For batch processing of species, while the circular buffer method does not take into consideration the dispersal abilities of each taxon, it was recommended by Rivers et al. (2010) after testing various methods. The output must be considered as an approximation of the total number of subpopulations. Although the number of subpopulations is not directly taken into account for assessments based on criterion B, this information is requested for the submission of full assessments to the IUCN Red List.

### 3.4 *map.res*

**Figure 2:**
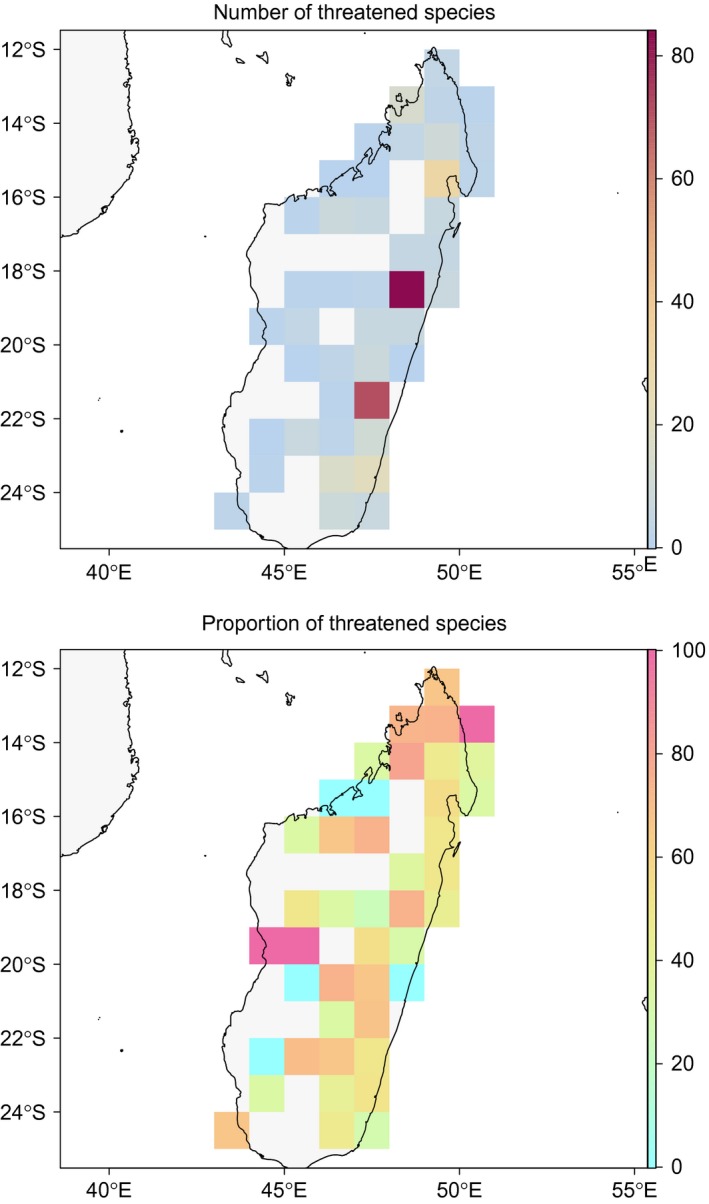
Two examples of map outputs generated by the *map.res* function. The dataset contains distribution information for 201 Malagasy amphibian species and 7,657 occurrence records (available from GBIF (https://doi.org/10.15468/dl.2tkoae) for Malagasy amphibians. (a) Total number of species assigned to one threat category by the *IUCN.eval* function per 0.5° sample units. (b) Proportion (in %) of threatened species per 0.5° sample units

The *map.res* function allows a graphical summary and geographical exploration of the results of the *IUCN.eval* function by generating maps with user‐specified unit sizes (Figure 2). These maps can show, for each unit, the number of records, number of taxa, number of threatened taxa, and proportion of threatened taxa.

## 4 CASE STUDY

In order to illustrate the usefulness and limits of *ConR*, we tested the package on a high‐quality dataset of continental African palm distributions for 60 species (of the 68 currently known; Stauffer et al., 2017). A large part of the data were extracted from the RAINBIO database (Dauby et al., 2016), which contains nearly all herbarium collections for African palms. Additional recent collections were added when available, resulting in a dataset of 4,234 unique occurrence records. The dataset was first used for the preparation and submission (as of April 2017) of full, species by species IUCN Red Listing assessments, mainly under Criterion B. Second, using *ConR* (with default parameters) and the same data for all 60 species, but excluding any “nonherbarium” occurrences (such as those based on satellite imagery or population censuses), we performed preliminary assessments as a batch operation. We also ran the dataset with and without PA information (downloaded and filtered from https://www.protectedplanet.net) using the “protect.areas” default option. *ConR* analyzed the dataset in less than 5 minutes using a standard laptop.

The results of the full IUCN assessments and those generated by *ConR* (with and without PA information), summarized in Table 3, are quite congruent. Factoring in PAs did not alter the outcomes, expect for a single species: *Oncocalamus wrightianus* was assessed as EN in the full assessment and by *ConR* when no PA information was included, but as VU with PA information. Regarding whether a species was assessed as threatened (i.e., CR, EN or VU) or not (NT or LC), we see that for 43 species (71%), the results from *ConR* and the full assessments agreed. For seven species, the *ConR* assessment indicated a threatened status, whereas the full assessment was LC. Finally, for eight species, the full assessment yielded a status of Data Deficient (DD) while *ConR* suggested either EN (5 species) or CR (3 species).

**Table 3:** Comparison of preliminary (*ConR*) versus full conservation assessments using a case study of Africa palm species (excluding Madagascar). *Hyphaene macrosperma*, only known from the type specimen, for which the location is very vague, was not included in the *ConR* analysis (indicated as NA in the table). For *Laccosperma cristalensis*, a single collection is known, but with precise coordinates, so this taxon was retained for *ConR* analysis but the EOO was not calculated (indicated as NA)

Species	Individual Red List assessment	Automatic Red List assessment *ConR* without PA information			Automatic Red List assessment *ConR* with PA information		
		Selected	Following B2 based on EOO	Following B1 based on AOO	Selected	Following B2 based on EOO	Following B1 based on AOO
*Borassus aethiopum* Mart.	LC B2	LC B1a + B2a	NT or LC	NT or LC	LC B1a + B2a	NT or LC	NT or LC
*Borassus akeassii* Bayton, Ouédr. & Guinko	LC B2	LC B1a + B2a	NT or LC	NT or LC	LC B1a + B2a	NT or LC	NT or LC
*Calamus deerratus* G. Mann & H. Wendl.	LC B2	LC B1a + B2a	NT or LC	NT or LC	LC B1a + B2a	NT or LC	NT or LC
*Elaeis guineensis* Jacq.	LC B2	LC B1a + B2a	NT or LC	NT or LC	LC B1a + B2a	NT or LC	NT or LC
*Eremospatha barendii* Sunderl.	CR B1ab(iii) + B2ab(iii)	EN B1a + B2a	EN	EN	EN B1a + B2a	EN	EN
*Eremospatha cabrae* (T. Durand & Schinz) De Wild.	LC B2	LC B1a + B2a	NT or LC	NT or LC	LC B1a + B2a	NT or LC	NT or LC
*Eremospatha cuspidata* (G. Mann & H. Wendl.) G. Mann & H. Wendl.	LC B2	LC B1a + B2a	NT or LC	NT or LC	LC B1a + B2a	NT or LC	NT or LC
*Eremospatha dransfieldii* Sunderl.	EN B2ab(iii)	VU B2a	NT or LC	VU	VU B2a	NT or LC	VU
*Eremospatha haullevilleana* De Wild.	LC B2	LC B1a + B2a	NT or LC	NT or LC	LC B1a + B2a	NT or LC	NT or LC
*Eremospatha hookeri* (G. Mann & H. Wendl.) H. Wendl.	LC B2	LC B1a + B2a	NT or LC	NT or LC	LC B1a + B2a	NT or LC	NT or LC
*Eremospatha laurentii* De Wild	LC B2	LC B1a + B2a	NT or LC	NT or LC	LC B1a + B2a	NT or LC	NT or LC
*Eremospatha macrocarpa* (G. Mann & H. Wendl.) H. Wendl.	LC B2	LC B1a + B2a	NT or LC	NT or LC	LC B1a + B2a	NT or LC	NT or LC
*Eremospatha quinquecostulata* Becc.	LC B2	VU B2a	NT or LC	VU	VU B2a	NT or LC	VU
*Eremospatha tessmanniana* Becc.	LC B2	EN B2a	NT or LC	EN	EN B2a	NT or LC	EN
*Eremospatha wendlandiana* Dammer ex Becc.	LC B2	LC B1a + B2a	NT or LC	NT or LC	LC B1a + B2a	NT or LC	NT or LC
*Hyphaene compressa* H. Wendl.	LC B2	LC B1a + B2a	NT or LC	NT or LC	LC B1a + B2a	NT or LC	NT or LC
*Hyphaene coriacea* Gaertn.	LC B2	LC B1a + B2a	NT or LC	NT or LC	LC B1a + B2a	NT or LC	NT or LC
*Hyphaene guineensis* Schumach. & Thonn.	LC B2	LC B1a + B2a	NT or LC	NT or LC	LC B1a + B2a	NT or LC	NT or LC
*Hyphaene macrosperma* H. Wendl.	DD	NA	NA	NA	NA	NA	NA
*Hyphaene petersiana* Klotzsch ex Mart.	LC B2	LC B1a + B2a	NT or LC	NT or LC	LC B1a + B2a	NT or LC	NT or LC
*Hyphaene reptans* Becc.	DD	EN B2a	NT or LC	EN	EN B2a	NT or LC	EN
*Hyphaene thebaica* (L.) Mart.	LC B2	LC B1a + B2a	NT or LC	NT or LC	LC B1a + B2a	NT or LC	NT or LC
*Laccosperma acutiflorum* (Becc.) J. Dransf.	LC B2	LC B1a + B2a	NT or LC	NT or LC	LC B1a + B2a	NT or LC	NT or LC
*Laccosperma cristalensis* Couvreur & Niang.	DD	CR B2a	NA	CR	CR B2a	NA	CR
*Laccosperma korupense* Sunderl.	LC B2	LC B1a + B2a	NT or LC	NT or LC	LC B1a + B2a	NT or LC	NT or LC
*Laccosperma laeve* (G. Mann & H. Wendl.) H. Wendl.	LC B2	LC B1a + B2a	NT or LC	NT or LC	LC B1a + B2a	NT or LC	NT or LC
*Laccosperma opacum* (G. Mann & H. Wendl.) Drude	LC B2	LC B1a + B2a	NT or LC	NT or LC	LC B1a + B2a	NT or LC	NT or LC
*Laccosperma robustum* (Burret) J. Dransf.	LC B2	LC B1a + B2a	NT or LC	NT or LC	LC B1a + B2a	NT or LC	NT or LC
*Laccosperma secundiflorum* (P. Beauv.) Kuntze	LC B2	LC B1a + B2a	NT or LC	NT or LC	LC B1a + B2a	NT or LC	NT or LC
*Livistona carinensis* (Chiov.) J. Dransf. & N.W. Uhl	EN B2ab(iii,v) A2ac	EN B1a + B2a	EN	EN	EN B1a + B2a	EN	EN
*Medemia argun* (Mart.) Württemb. ex H. Wendl.	VU B2ab(iii)	LC B1a + B2a	NT or LC	NT or LC	LC B1a + B2a	NT or LC	NT or LC
*Oncocalamus macrospathus* Burret	LC B2	LC B1a + B2a	NT or LC	NT or LC	LC B1a + B2a	NT or LC	NT or LC
*Oncocalamus mannii* (H. Wendl.) H. Wendl.	LC B2	LC B1a + B2a	NT or LC	NT or LC	LC B1a + B2a	NT or LC	NT or LC
*Oncocalamus tuleyi* Sunderl.	LC B2	LC B1a + B2a	NT or LC	NT or LC	LC B1a + B2a	NT or LC	NT or LC
*Oncocalamus wrightianus* Hutch. & H. Wendl.	EN B1ab(iii) + B2ab(iii)	EN B1a + B2a	EN	EN	VU B1a + B2a	VU	VU
*Phoenix caespitosa* Chiov.	LC B2	VU B2a	NT or LC	VU	VU B2a	NT or LC	VU
*Phoenix reclinata* Jacq.	LC B2	LC B1a + B2a	NT or LC	NT or LC	LC B1a + B2a	NT or LC	NT or LC
*Podococcus acaulis* Hua	LC B2	LC B1a + B2a	NT or LC	NT or LC	LC B1a + B2a	NT or LC	NT or LC
*Podococcus barteri* G. Mann & H. Wendl.	LC B2	LC B1a + B2a	NT or LC	NT or LC	LC B1a + B2a	NT or LC	NT or LC
*Raphia africana* Otedoh	DD	EN B2a	NA	EN	EN B2a	NA	EN
*Raphia farinifera* (Gaertn.) Hyl.	LC B2	LC B1a + B2a	NT or LC	NT or LC	LC B1a + B2a	NT or LC	NT or LC
*Raphia gentiliana* De Wild.	LC B2	VU B2a	NT or LC	VU	VU B2a	NT or LC	VU
*Raphia hookeri* G. Mann & H. Wendl.	LC B2	LC B1a + B2a	NT or LC	NT or LC	LC B1a + B2a	NT or LC	NT or LC
*Raphia laurentii* De Wild.	LC B2	VU B2a	NT or LC	VU	VU B2a	NT or LC	VU
*Raphia longiflora* G. Mann & H. Wendl.	DD	CR B2a	NA	CR	CR B2a	NA	CR
*Raphia mambillensis* Otedoh	LC B2	LC B1a + B2a	NT or LC	NT or LC	LC B1a + B2a	NT or LC	NT or LC
*Raphia mannii* Becc.	DD	CR B2a	NA	CR	CR B2a	NA	CR
*Raphia matombe* De Wild.	DD	EN B2a	VU	EN	EN B2a	VU	EN
*Raphia monbuttorum* Drude	LC B2	VU B2a	NT or LC	VU	VU B2a	NT or LC	VU
*Raphia palma‐pinus* (Gaertn.) Hutch.	NT B2b(iii)	LC B1a + B2a	NT or LC	NT or LC	LC B1a + B2a	NT or LC	NT or LC
*Raphia regalis* Becc.	LC B2	LC B1a + B2a	NT or LC	NT or LC	LC B1a + B2a	NT or LC	NT or LC
*Raphia rostrata* Burret	DD	EN B1a + B2a	EN	EN	EN B1a + B2a	EN	EN
*Raphia ruwenzorica* Otedoh	DD	EN B2a	VU	EN	EN B2a	VU	EN
*Raphia sese* De Wild.	LC B2	LC B1a + B2a	NT or LC	NT or LC	VU B2a	NT or LC	VU
*Raphia sudanica* A. Chev.	NT B2b(v)	LC B1a + B2a	NT or LC	NT or LC	LC B1a + B2a	NT or LC	NT or LC
*Raphia textilis* Welw.	LC B2	EN B2a	NT or LC	EN	EN B2a	NT or LC	EN
*Raphia vinifera* P. Beauv.	LC B2	LC B1a + B2a	NT or LC	NT or LC	LC B1a + B2a	NT or LC	NT or LC
*Sclerosperma mannii* H. Wendl.	LC B2	LC B1a + B2a	NT or LC	NT or LC	LC B1a + B2a	NT or LC	NT or LC
*Sclerosperma profizianum* Valk.	LC B2	LC B1a + B2a	NT or LC	NT or LC	LC B1a + B2a	NT or LC	NT or LC
*Sclerosperma walkeri* A. Chev.	LC B2	LC B1a + B2a	NT or LC	NT or LC	LC B1a + B2a	NT or LC	NT or LC

In addition to this case study, we also undertook a preliminary conservation assessment of amphibians of Madagascar using a dataset that contained 7,657 georeferenced records representing 201 species, downloaded on February 9, 2016, from www.gbif.org (https://doi.org/10.15468/dl.2tkoae). This analysis was performed mainly to demonstrate the graphical outputs of *ConR* (Figures 1 and 2). This dataset is available within *ConR* as an example data frame (Malagasy_amphibian).

## 5 DISCUSSION

*ConR* provides for the first time a dedicated, multispecies conservation assessment package based specifically on IUCN criterion B and using only species geographic distribution. It provides an efficient tool to help accelerate the work of the conservation community by enabling practitioners to conduct preliminary assessments that are both reliable and informative. We stress that *ConR* does not (and is not intended too) replace the full IUCN Red Listing process; it can, however, assist and facilitate this process. *ConR* uses a number of assumptions in order to automate category assignment, especially the estimation of the number of locations *sensu* IUCN. Notwithstanding these assumptions, detailed above, which must be understood and acknowledged by the user, *ConR* is flexible in their implementation, allowing the user to explore various approaches and methodologies and to customize values for each option. As shown in our case study on African palms (Table 3), the results of the full and *ConR* assessments are generally congruent. The differences observed between them can be linked primarily to the way in which *ConR* estimates the number of locations. For example, *Eremospatha barendii* is known from three collections made at localities more than 10 km apart. *ConR* thus infers (with a resolution of 10 km^2^) two locations, whereas in the full assessment, we estimated a single location (because both localities were considered to be subjected to the same threat). Another difference lies in whether locations were used or not for the full assessment. For example, *Eremospatha dransfieldii* is inferred by *ConR* to have eight locations, and it is therefore assessed as VU. However, the subpopulations of this species are severely fragmented (Cosiaux et al., 2017), which also triggers subcriterion “a” (Table 1), which was used along with continuing decline (subcriterion “b”), and thus the number of locations was not used for the full assessment. In contrast, for some species, *ConR* indicated a status of CR, EN or VU, whereas the full assessment was LC (e.g., *Raphia gentiliana* and *R. monbutturom*; Table 3). These mismatches occurred for species with broad geographic distributions but for which there were few collections (and thus fewer than 10 locations were inferred by *ConR*), while field data provided no clear evidence of highly fragmented populations.

This case study clearly illustrates that *ConR* (1) can be used to generate fairly reliable preliminary conservation assessments on large datasets, but (2) has limitations when, for example, a species is widespread and common but poorly collected or is widespread but severely fragmented. It is important to stress that the accuracy of the georeferencing in such datasets is crucial for estimating risk. Two recently released R packages, *Biogeo* (Robertson, Visser, & Hui, 2016) and *speciesgeocode*R (Töpel et al., 2017), are designed to help curate and clean large datasets, and are thus complementary to *ConR*.

*ConR* will be useful for a variety of applications. First, as distributional data for taxa become increasingly available via large multitaxon databases (Dauby et al., 2016; Marshall, Wieringa, & Hawthorne, 2016; see also Botanical Information and Ecology Network http://bien.nceas.ucsb.edu/bien/) and online repositories (Global Biodiversity Information Facility, http://www.gbif.org/; Atlas of Living Australia; www.ala.org.au), *ConR* makes it possible to calculate/estimate key parameters for conducting preliminary assessments of conservation status based on selected IUCN criteria and to provide data on the threat of multiple species for a region, a specific clade, a functional group, etc. The application of *ConR* to large datasets could also contribute to meeting Target 2 of the Global Strategy for Plant Conservation (GSPC).

Second, *ConR* will also be of value to taxonomists, who are increasingly expected to provide preliminary conservation assessments when describing new species or publishing revisions or monographs. By generating key parameters (EOO, AOO and an estimate of the number of locations), *ConR* will greatly facilitate this process.

Finally, rapid preliminary assessments of IUCN conservation status based on large, multitaxon sets will support studies on a wide range of subjects such as the evolution of extinction risk within and among clades (Forest, Crandall, Chase, & Faith, 2015; Jetz et al., 2014) and the phylogenetic component of extinction risk within regional floras (Leão, Fonseca, Peres, & Tabarelli, 2014) or faunas.

The *ConR* package has already been used by the authors to facilitate full assessments (e.g., of palms) and to prepare IUCN Red List workshops. Also, *ConR* has been successfully used as part of an IUCN “Green Listing” of Protected and Conserved Areas (IUCN, 2012) for private sector players in order to identify potentially threatened species occurring in their concessions that, after verification, will be the subject of specific conservation management plans (unpublished results).

## DATA ACCESSIBILITY

The *ConR* package is written in R (R development Core Team 2016) and is available on the Comprehensive R Archive Network (https://cran.r-project.org/package=ConR) and on a github repository (https://github.com/gdauby/ConR).

## CONFLICT OF INTEREST

None declared.

## AUTHOR CONTRIBUTIONS

TLPC, TS, PPL, GES, REG and GD conceived the study; GD wrote the package; AC and TLPC analysed the palm data; MSD, VDr, MSMS, AC, TS, GD, VDe and TLPC tested the package; and all authors contributed to writing the article.
